# Achieving professional status: Australian podiatrists' perceptions

**DOI:** 10.1186/1757-1146-2-4

**Published:** 2009-02-13

**Authors:** Alan M Borthwick, Susan A Nancarrow, Wesley Vernon, Jeremy Walker

**Affiliations:** 1 School of Health Sciences, University of Southampton, Highfield, Southampton, UK; 2 Senior Research Fellow, Centre for Health and Social Care Research, Faculty of Health and Wellbeing, Sheffield Hallam University, Sheffield, UK; 3 Department of Podiatry, Sheffield South West Primary Care Trust, Sheffield, UK

## Abstract

**Background:**

This paper explores the notion of professional status from the perspective of a sample of Australian podiatrists; how it is experienced, what factors are felt to affect it, and how these are considered to influence professional standing within an evolving healthcare system. Underpinning sociological theory is deployed in order to inform and contextualise the study.

**Methods:**

Data were drawn from a series of in-depth semi-structured interviews (n = 21) and focus groups (n = 9) with podiatrists from across four of Australia's eastern states (Queensland, New South Wales, Victoria and Australian Capital Territory), resulting in a total of 76 participants. Semi-structured interview schedules sought to explore podiatrist perspectives on a range of features related to professional status within podiatry in Australia.

**Results:**

Central to the retention and enhancement of status was felt to be the development of specialist roles and the maintenance of control over key task domains. Key distinctions in private and public sector environments, and in rural and urban settings, were noted and found to reflect differing contexts for status development. Marketing was considered important to image enhancement, as was the cache attached to the status of the universities providing graduate education.

**Conclusion:**

Perceived determinants of professional status broadly matched those identified in the wider sociological literature, most notably credentialism, client status, content and context of work (such as specialisation) and an ideological basis for persuading audiences to acknowledge professional status. In an environment of demographic and workforce change, and the resultant policy demands for healthcare service re-design, enhanced opportunities for specialisation appear evident. Under the current model of professionalism, both role flexibility and uniqueness may prove important.

## Background

Acquiring, securing and elevating professional status is a central aim for many health care professions, which constantly seek ways of preserving and enhancing opportunities for higher social status and recognition [1,2]. Furthermore, within the health division of labour, there is a hierarchy of relative power and prestige characterised by a medical hegemony around which a range of 'allied health' professions operate within limited boundaries, including the podiatry profession [3-6]. Whilst an expansion in scope of practice, a growth in higher educational attainment and successful legislative closure strategies have characterised the relatively recent past in the profession of podiatry in both Australia and the UK, problems of recruitment, retention and reported 'burn-out' continue to be attributed, in part, to low social status [7-9].

Yet, evidence of change has emerged from studies in the USA. Chumbler and Grimm [10-12], Grimm and Chumbler [13] and Chumbler and Brooks [14] reported an upward trend in self-perceptions of professional status among podiatrists in the USA, following the acquisition of hospital medical-staff privileges. Increased demand for podiatry, attributable to demographic ageing and 'recreational' trends (such as jogging), alongside greater sophistication in technological advances (such as invasive surgery and 'biomechanics') were felt to be important determinants of change [11]. However, these demographic and technical developments are also evident in the UK and Australia, with less evidence of improvements in self-esteem or professional status [7,15,16].

It has been noted that service expansion and recruitment within podiatry has increased in Australia over the last decade, in sharp contrast to the UK, where an acute shortage of recruits has been prevalent for some time [16-19]. Whilst a recent account of perceived professional status within podiatry provides insights into professional self-perceptions in the UK profession [9], this study seeks to gain a similar, in-depth, insight into professional's perceptions in Australian podiatry.

This study was given extra impetus by the current context of health policy reform in both the UK and Australia, which has emphasized professional role boundary change as a way to increase workforce flexibility, overcome staff shortages, and potentially enhance workforce productivity [20,21]. This policy support may be seen as a potential resource in the struggle for increased professional status for the 'allied' health professions in an era of increasing independence from medical control [22,23]. Yet some key health policy documents view certain 'workforce behaviours', where 'cultural attitudes can reinforce notions of high status', as obstacles to workforce flexibility and planning [24].

In the analysis presented below, podiatrists' accounts of their own professional status, and their understanding of the factors which might influence their status, are adopted in favour of repeating prior work utilising occupational prestige rating scores or measures of 'burn-out'. Similarly, Chumbler and Grimm's [25] status enhancement scales are limited in that they focus on inter-professional referral networks in establishing perceptions of relative status. In this study, perceptions of professional status within podiatry in Australia were not confined to ratings of prestige between occupations, or linked specifically to interpersonal ties between podiatrists and physicians, but explored through in-depth interviews in a bid to gain a deeper insight into the way in which the respondents viewed their professional status; to reveal the extent to which they felt their status might be influenced, positively or negatively, and what strategies they considered important in enhancing it.

### Podiatry in Australia

Australian podiatry labour force data indicates that there were 2,361 registered podiatrists in Australia in 2003, compared to 10,123 in the UK [26]. Interestingly, whilst in the UK the majority of podiatrists work in the public sector (46.7%), in most States in Australia the percentage is much lower, ranging from 35% in Victoria to around 15% in New South Wales [16,17].

As elsewhere in the Anglophone world, podiatry in Australia evolved within a hierarchical division of labour characterised by the hegemonic position of medicine [23,27,28]. Whilst Australian podiatry first attained legislative recognition as an independent profession in 1950 [29], it remained, as did other non-medical healthcare professions, subject to the forces of medical authority and 'sovereignty' [5,28]. In common with the profession in other Commonwealth countries, podiatry in Australia was originally closely modelled on its British counterpart, and the introduction of a uniform three year training programme in the late 1960s established an educational equivalence which remains broadly extant [27,30,31]. Nevertheless, there are subtly different influences relevant to individual states within the federal nation which exist in relation to factors such as client groups, state funding arrangements, practitioner numbers relative to population and age profiles [17,32].

### Professional status: theoretical considerations

Professional status is usually considered in the literature to be a by-product of successful market strategies, and as a reward granted by society for high educational achievement or for other significant attainments which reflect important societal values [2,33]. In order to give meaning to the way in which the respondents understood their professional status (as a variable over which they might command some control), an examination of current theoretical approaches to the concept of professional status is undertaken to inform the findings.

Within the Weberian tradition, social status may be either 'ascribed' or 'achieved' [1,34,35]. Ascribed status, over which individuals are able to exert little influence (when based on race or gender, for example), is firmly associated with pre-modern societies, whereas 'achieved' status is viewed as a characteristic of contemporary societies and marked by an emphasis on individual social mobility [2,34,36]. Occupations are widely regarded as one key source of ascribed social status, most clearly evident within the category of professional occupations, where members share certain privileges in line with their assigned place in the hierarchy of esteem [2,34-37].

Whilst Larson [1], Parkin [38] and Turner [34], among others, focus to a large extent upon education as the principle means to secure 'achieved' social status in the professions, other factors are also viewed as important. Macdonald [36] and Collins [39], for example, stress the importance to professions of persuading key audiences – such as the State, the public and other professions [36]. Symbolic displays of respectability or the use of an 'ideological covering' contribute towards status enhancement [39,40].

Abbott [41] examined the premise that wealth, power, client status and non-routine work were important determinants of high status in the professions, but found exceptions in each case. In addition, he noted that the public tended to rank 'front-line professionals' most highly, in contrast to the professionals, who ascribed greater prestige to professional leaders or academics. For Leicht and Fennell [40], lower status is ascribed to those working with 'stigmatised populations', identified specifically as comprising patients who are 'dangerous, lacking in resources and/or socially outcast'. This 'medical equivalent of "dirty work"' [40] has parallels within nursing and the allied health professions, where 'ditching the dirty work' commonly involves minimising social contact with low status service user groups such as the elderly and those demanding longer term care and support (rather than those requiring acute interventions directed towards curing and problem-solving) [4,6]. High status work is said to comprise that in which sophisticated technologies or skills demanding specialised knowledge are deployed [40,42]. Hugman [4] identified a trend within the allied health professions towards emulating those sorts of medical practices through the development of 'virtuoso' roles, through which higher professional status is attained. Virtuoso roles are deemed to 'have glamour', to 'carry effective autonomy, and produce measurable and observable outcomes [cure as opposed to caring]' [4].

## Methods

Status is considered in this paper as a subjective evaluation, following the analysis of Lipset [43] who defined status as 'the positive or negative estimation of honour, or prestige received by individuals or positions. Thus, it involves the felt perceptions of people'. This view is also reflected by Turner [34], who suggested that 'prestige is a social-psychological category: an individual or social group cannot enjoy it unless their prestige claims are recognised by others willing to give them deference', echoing Veblen [44], who declared that 'the usual basis of self-respect is the respect accorded by one's neighbours'. It was not felt appropriate to employ a quantitative indicator of occupational status based on synthetic comparative ratings of podiatry in relation to other professions. Rather, this study sought to explore the respondents' perceptions of status and the role and relevance of factors such as client type, content of work, educational development and the impact of relations with medicine.

The data in this study were derived from a series of in-depth, key informant, focus group and telephone interviews with stakeholders. Respondents were drawn from the Eastern seaboard states of New South Wales (Sydney and Newcastle), Australian Capital Territory (Canberra), Victoria (Albury and Melbourne) and Queensland (Brisbane). Participants were initially recruited via a flyer which was circulated through the professional associations within each of the participating states, as well as through academic contacts at the respective universities in each city (except Newcastle, which did not have training facilities for podiatrists at the time of undertaking the research). According to the Australasian Podiatry Council, more than 80% of practicing podiatrists are members of the professional association, which means that respondents recruited this way are more likely to be representative of the majority of Australian podiatrists [45]. Overall twenty-one semi-structured interviews and nine focus group interviews were conducted, resulting in a total participation of 76 podiatrists. The method of recruiting participants meant that sampling was, initially, largely opportunistic. However once we had made contact with the initial participants, we were able to use snowball sampling techniques to purposively sample further participants in a bid to ensure largely representative coverage of specific professional sub-groups, and ensure diversity in terms of rural and urban practitioner responses.

To protect the identity of the participants, their details have not been tabulated; however a broad breakdown of their characteristics follows. Of the 21 individual interviews, 10 participants were female and 11 were male. Five participants were from Queensland, eight were from NSW and eight from Victoria. Five of the participants were actively involved either in their state professional association (including Victoria, NSW and Queensland representatives), or the Australian Podiatry Council (the national professional organisation representing podiatry in Australia). One podiatric surgeon and five academic podiatrists were interviewed. All but five of the podiatrists worked primarily in private practice. Eight of the interviewees worked in rural areas. Two were based in urban areas and undertook outreach work into rural areas.

The nine focus groups were performed in Sydney (two), Newcastle, the Australian Capital Territory, Melbourne (two), Albury and Brisbane (two). A total of 39 podiatrists participated in the focus groups, including 22 males and 17 females; nine academic podiatrists (some of whom also worked in the public and private sector part-time). Six of the participants worked solely in the public sector, and the remainder were private practitioners.

Research governance approval was obtained through the Sheffield Health and Social Care Research Consortium. At the time of undertaking the research, there was no appropriate jurisdictional body for ethical approval for research undertaken through a professional body, however approval and support was obtained by the Podiatry Associations in each state, and every participant was provided with a participant information sheet, and asked to sign a consent form.

The interviews were conducted by all the authors using a standardised interview schedule which explored the scope of practice of podiatry; participants' perceptions of the status of podiatry generally, and in relation to other health care professions; the relative status of different types of podiatry work; factors that influence the status of the profession positively and negatively; career development opportunities, interprofessional working and the introduction of support workers. The themes arose directly from the research questions and were informed by current policy issues (in particular, the issues around interprofessional working and the introduction of new workers), and the published literature.

All interviews were tape recorded and transcribed verbatim. The members of the research team undertook thematic analysis of the transcripts using the Ritchie and Spencer Framework approach [46] which involves the systematic familiarisation of the data; identifying a thematic framework; indexing the themes; charting those themes into a hierarchical framework; then mapping and interpreting the themes.

Transcripts were coded using a priori constructs arising from the interview theme list, and with the addition of new themes arising from the data. All of the team read the transcripts and were involved in the formation of the thematic framework, indexing and development of the hierarchical structure. The resulting structure was mapped onto the transcripts. All of the research team were involved in the subsequent analysis.

To verify the findings, two of the original interview participants were sent a copy of the manuscript and asked to comment on the extent to which they perceived the findings accurately represented their perception of the issues affecting status in Australia. Both provided feedback which has been incorporated into the paper.

## Results

### The impact of specialised roles on status

Most respondents considered that their professional status had grown in recent years, and drew upon a number of factors to account for this perception. Most commonly, expanding role boundaries and the acquisition of more 'specialised' skills were felt to have enhanced professional status both in the eyes of the public and other related professions. Enhanced public visibility in areas of practice associated with more glamorous roles was also felt to be important. Indeed, the transition from the image of 'corn-cutters' [47] to specialists in 'lower limb biomechanics' was felt to be an indicator of a shift in public attitude, although 'corns and calluses' still constituted an inescapable core element of practice [18,19].

*It [professional status] seems to have improved greatly over the last number of years. I quite happily tell people now that I'm a podiatrist. I used to say I'm a corn merchant or I work in public health or something like that, you know, because of the fact that often people say 'oh, I've got this thing on my foot, look at this'. People tend to be more aware of what a podiatrist is, perhaps, and what a podiatrist does, you know? Not 'Oh, you cut people's toenails and you trim people's corns'. People seem to have more awareness of the fact that podiatrists can look at other aspects of foot health, biomechanics...I think it's a lot to do with...having a larger scope of practice, I think tends to raise the status because you tend to get involved with more branches of medicine... [but] I know that a lot of males particularly 'burn out' badly in the first five years because of that status thing. It's not what they thought it was going to be, you know, the bread and butter still exists off the corns and the calluses and the toenails... *(Metropolitan Focus Group, New South Wales)

Specialist work, primarily undertaken in the private sector, was viewed as instrumental in enhancing professional status – most notably in the arena of 'sports'. It was felt that this area permitted a reconfiguration in service provision within a market characterised by young client groups. Association of podiatry with sports injuries care and prevention was viewed as both a lucrative market opportunity as well as a means of establishing greater social status. Indeed, in Queensland there was a noticeable student recruitment trend from other Human Movement courses (which had no clearly associated career pathway) which was related to perceptions of podiatry as a specific pathway which would enable a career in sports and athletic health care.

*We changed the podiatry degree two years ago [in Brisbane] to make sure the programmes were attractive...quite a number of those [newly recruited students]...were interested in becoming podiatrists [because] they all thought that they would become better at biomechanical therapy thereby making better sports medicine practitioners, that is [sic] their mindset. Human movement studies here is perhaps similar to elsewhere and does not have a career path...there isn't a defined career, job, profession, in the end. So they look at other options...about 30% of our enrolment consignees have been...students who have already completed a movement studies degree...the human movement schools, of which there are 3 in Brisbane, alone have a student intake of 300 intakes per school [per year]. So there's...students...each year get to the end and there is no career path. *(Interview with Queensland Podiatrist)

Indeed, the respondents viewed Australia as a nation with a young population culturally attached to sports. By establishing a link between sport, sports injuries and podiatry, the profession was felt to have advanced its status with the public; the focus on recruiting a client base of young, athletic individuals was felt to be crucial in the bid to attain greater status.

*There is such a young population...the 10% who are doing that, the sports podiatry...it is a sporting country...sport is very available in a rural setting...there is every code of sport embraced that you can think of...the sports factor is increasing the number of sports injuries...and podiatry is seen as one of the possible ways identifying the problem or curing the problem...It is the big sports factor, which is leading the kids to find out more about podiatry. *(Rural Focus Group, New South Wales)

However, physiotherapy was seen as rather more successful in cornering a market in sports care, having established roles in professional sports clubs and representation in the Australian Institute of Sport.

*There is glamour associated with physiotherapy...I don't think podiatry has any glamour whatsoever...Every sports team has got a physio on it [sic]. Any of your famous sports stars have seen their physio recently for their foot [problems]...also, physios are at the University of Sydney [which] is the best, the highest medical [education]...You need key people in 'high faluting' positions such as AIS [Australian Institute of Sport] type podiatrists... [currently] there's no such thing...They don't employ a podiatrist, they employ a physiotherapist... *(Metropolitan Focus Group, New South Wales)

Other, more recently acquired, aspects of practice were considered to have raised the status of podiatry in the eyes of similar professional groups, as these techniques were seen as more overtly 'medical' and technically complex. In addition, patient expectations of the role of the podiatrist were felt to be changing, enabling the podiatrist to assume a more consultative role, offering advice and knowledge, rather than engaging purely in labour intensive and relatively simple tasks carried out at the direction of the patient.

*Interestingly podiatrists can break the skin surface, which people like physiotherapists think is absolutely fantastic, because they can't. So things like being able to do...surgical procedures tends to raise our status among a lot of other allied health professionals that aren't allowed to pierce the skin in any shape or form...Similarly, being able to refer for things like x-rays...the patients have a larger expectation in recent years than they did 20 or 30 years ago...I mean, I will spend the consultation like this [indicates], with my arms folded, talking about things, without actually touching them, you know...giving them advice...footwear...sports opinions. Whereas 20 years ago it was very much, you know...'I have this problem with this toenail, fix it up, I'll read the newspaper while you do it'...like 'well, I go to the barber to get my hair cut and he talks about the weather and I just tune out, and I go to the chiropodist and I get my corns cut'... It was...not so much a health but a body maintenance situation, and I think that perception is changing. *(Metropolitan Focus Group, New South Wales)

It was also the case that some respondents suggested that practitioners often claimed 'specialist' status when only undertaking a small amount of such work, in order to bolster professional status amongst peers as well as patients.

*We ran a biomechanics business information programme and...I ran...the first project that... [did] a demographic analysis of the practices. It is always interesting having those people coming in saying 'I am a biomechanics specialist' – you actually get them to do a demographic analysis for what they see on a day to day basis and they are seeing less than 10%. Less than 10% of their practice is actually what they perceive as their specialisation. In paediatrics it is less than 2%. What that means is that they remember the thing that is most sexy, most exciting...they are not actually seeing the numbers of people that they should be seeing *(Rural Focus Group, New South Wales)

### Exclusivity of task domains

The role and task domain of the podiatrist was consistently viewed as central to the maintenance and enhancement of professional status, alongside the extent to which these tasks or roles were exclusive to the profession. Where other professional groups were perceived as having successfully breached these role boundaries, or successfully ensured exclusion from others, the status of the profession was deemed to have suffered.

*I think...the curse that podiatrists have put upon themselves by legislating themselves to a limitation, which is foot and ankle. We have many other professions, such as chiropractors or physiotherapists, and now also the nurse practitioners, who can treat the whole body and who are also starting to develop into podiatry...podiatry has a history of relinquishing aspects of its practice. For instance, biomechanics was regarded as a podiatric cousin of medicine for a period of time...now we have physiotherapists that do biomechanics, we have chiropractors that do biomechanics, we have doctors that do biomechanics and sports clinics and so on. Now foot biomechanics can also be practised by nurse practitioners...They will be able to do everything a podiatrist can do. *(ACT Focus Group)

*Well, if we compare ourselves to other medical practitioners, so to speak, then not being able to prescribe simple podiatric necessary medications is, I think, a big thing in terms of our status...why is it a person goes to a doctor to get their fungus in the nail sorted out, and not a podiatrist? Because a doctor can prescribe Lamisil [antifungal treatment], which they advertise on TV and say 'go and see your doctor'. Podiatrists can't actually prescribe it. So things like that are going to keep us down there ... *(ACT Focus Group)

### Public versus private provision of services

Distinctions between public and private sector work in each of the states featured prominently, as did the relative status of urban and rural practice locations. Whereas the content of work and client groups were identified as quite similar, the relative level of status perceived within the work sector and practice location did appear to differ. For example, within New South Wales, public sector podiatrists working in hospital settings were perceived as enjoying higher professional status than private sector practitioners, although the latter could expect a significantly higher income. This was explained in terms of the level of responsibility attached to the 'specialist' work of the hospital podiatrist, who would focus upon the treatment of limb-threatening disorders (such as the complications arising from diabetes) alongside the clear association of working 'with' medical staff in a distinctly medical arena. Private sector workers, on the other hand, viewed themselves as enjoying greater financial rewards within essentially business-oriented practices, but with less demands in terms of 'high risk' patient care and complexity of technical work.

*I mean, the public sector [podiatrists] in New South Wales...you get a top rate of $50,000...I'm blowing my own trumpet, but I mean I haven't earned less than $70,000 over the last ten years in private practice, and I'm not really slaving away...I wouldn't want to be working all the time in a high risk [hospital] foot clinic. I mean it can be quite exhilarating but it's bloody hard work. *(Metropolitan Focus Group, New South Wales)

*My first year [in private practice] was like, $80,000, it was ridiculous. I was like 'this is obscene, how can I be earning this much money when my friends working in the hospital are earning $35,000? This crazy, so for anyone with a desire for money whatsoever, they're not going to get into the public system. *(Metropolitan Focus Group, New South Wales)

### Rural versus urban provision of podiatry services

In rural settings, greater opportunities for enhancement of professional status were felt to exist because of the reduced accessibility or relative lack of availability of healthcare staff. For example, where a dearth of medical services existed, the podiatrist was increasingly established as a 'front-line' health professional, assuming many of the roles that would normally be undertaken by the doctor or nurse. Thus, the scope of practice was extended, allowing the podiatrist to assume a higher profile role in healthcare delivery in rural communities in a way which did not exist in more urban locations.

At the same time, specific difficulties were identified with the scope of practice in rural settings. Although podiatrists might take on roles from doctors or nurses due to a lack of supply of these professionals in rural areas, similarly the opportunity to practice specialist podiatric skills was limited due to lack of demand, because of low population levels in rural communities.

*The biomechanical side of things definitely [has greater status]...You get the people who are considered the sports podiatrists [practising biomechanics], which is a bit of a buzz name, because basically we're all sports podiatrists – we all do the same training...But down here [rural practice] I'm the only one... [there are] no specialists in rural areas, nah, you've got to travel up to the main city centres before you get into any of that. There's just no population here to support it...if I wanted to be a diabetes specialist podiatrist, I'd have half a day's work a week *(Interview with Rural practitioner, New South Wales)

Nevertheless, the absence of specialists meant that the rural practitioners were often asked to carry out specialist tasks by the patients, as referral to specialists invariably involved long journeys to urban centres.

...*if you want to refer someone, it's a two hour drive to get there, so they try and do everything themselves because the patients say: 'look, do I really have to? Can't you just do it?' *(Interview with Rural practitioner, New South Wales)

Public funding in rural communities was felt to be poor, with repercussions for practitioners working in the rural public sector. Many were thought to experience burn-out and feelings of loss of control associated with the burden of over-demand, impacting on a sense of status.

...*in certain rural areas in the public sector there is never enough money. They are seriously under funded, for instance, in Wagga, (Wagga Wagga is the largest populated centre located in the interior of NSW) where we have nearly 60,000 people, the public purse runs to podiatrists for two days a week. Now, we did have five, and we are back to two for that huge population...we service probably 120,000 people...Wagga has no diabetic or high risk [service]. So the perception that I am probably guilty of having is that these poor bastards, they have to work in awful conditions and there is not enough money and how can they possibly really get any kind of fulfilment in their shoving people through the system *(Rural Focus Group New South Wales)

### Audiences: the impact of external attitudes

Although broadly viewed as in transition, public perceptions were still considered to constitute a hurdle to be cleared, with several respondents remarking on the negative way their roles and status were construed by patients.

*Often in the public setting it was 'I've got my routine six or seven week return [appointment] and I'm paying six dollars fifty – here you go, love' and they give you a lolly! That's kind of, 'this is your worth, this is your value'... 'I'm 65 and I'm entitled'...I've that comment [sic] 'I'm doing you a favour, love, I'm keeping you in employment' *(interview with Metropolitan Focus Group, New South Wales)

However, an ascription of lower status was felt to be especially evident within the elderly patient group, where the perceived technical and educational developments in the profession were less likely to be granted credibility. For example, one podiatrist who had been assigned to the Australian Olympic team expressed frustration at the reaction of one elderly patient to this news.

*Mrs J, who had been coming in [to the practice] for the last twenty years, and you know, I went to the Olympics as one of the [official] podiatrists working there, and she saw the PR, the article done on me [sic] in the local newspaper...a sort of 'local boy does good' [sic]...and she said 'Ah, I see you [were] going to cut the toenails of all the athletes. *(Interview with rural podiatrist, Victoria)

In NSW the professional status of public sector podiatry was felt to have been reduced by the low visibility of the profession to service purchasers, who had permitted the role of the podiatrist to be subsumed by other professions, most notably nursing. This was compounded by the fact that nurses were thought to be better paid than podiatrists in the public sector. Such an apparent anomaly was viewed as an illustration of the erosive effects on professional status of public sector health policy decisions driven by economic considerations. The purchasing authorities had chosen to delegate podiatric tasks to nurses as an adjunct to their workload rather than employ podiatrists.

*Its easier to tag a few hours of foot care onto nurses duties... [which was seen as] better than paying a podiatrist, even if you could get one...nurses have a wider range of skills than podiatry [so its] more practical to add footcare duties to their role. Employers train nurses to do footcare so they can say 'oh well, we don't have to worry about the podiatrist any more... *(interview with public sector podiatrist in New South Wales)

### Role of marketing and self-promotion

Podiatry was felt to enjoy the highest status in Victoria, where there was also a greater number of public sector podiatrists. Public sector work was viewed as well developed in Victoria compared to other states. This perceived higher status was attributed to a more active professional body, greater public understanding of what podiatrists do, and wider marketing, enabled by the greater resources of the professional association in states with higher population densities and therefore professional membership. For areas of relatively low population density and of vast geographical size, such as Queensland, resources were more problematic, limiting the marketing ambitions of the state associations.

*They've got higher populations down in Victoria and New South Wales so obviously they can look at greater membership, therefore their associations have more funds with which to work with to increase their profile over a smaller geographical area, whereas for us it's very difficult. *(Queensland APA)

Self-promotion was also viewed as an important determinant in establishing, or changing, professional status. In most instances, this related to the content of work and the extent to which clients were aware of the range of skills and services offered by the podiatrist. In one account, described by a private practitioner in New South Wales, the experience of finding a local competitor in the private sector profiting from claims to be a 'specialist' in the field of 'biomechanics', led patients to assume that he was 'only' capable of providing 'lower level' work, and therefore meriting a lower status.

*But the status can vary enormously depending on how you promote yourself. Like some patients can attend routinely...six or eight weeks and they just let it slip 'oh, I happen to get my orthotics made by the specialist down at the sports medicine centre, but I come to you for the regular corn'. And I say, 'oh, by the way, we actually do the same thing. It's the same qualification. It's no more specialised. There is no other course. We have qualified with the same degree, it's just that one person might do it all day'... But, because they have only attended you for the one reason and perhaps they've never mentioned it to you before...they may not realise that you are capable of providing that treatment*. (Metropolitan Focus Group, New South Wales)

### Gender and status

Recent labour force survey data has shown that the proportion of males in the podiatry workforce increased slightly between 1999 and 2003 from 34.3% to 37.4%[17].

However, gender was seldom remarked upon as a relevant issue, although it did surface when respondents addressed the problem of coping with the labour intensive nature of non-specialist work, which was felt to be boring, low status and likely to lead to 'burn-out'. In this instance, women were viewed as enjoying the options of moving to part-time work and 'taking' employment breaks to have a family, which were seen as unavailable to males, who had to find other strategies to contend with the problems.

*That five year burn out is a big thing, if you've managed to get to five years. 30 years ago the profession was predominantly female. The burn out didn't quite happen in the same way, because there was more of that traditional thing of people leaving work to have babies and raise families, at least for a few years, and then going back to work and the mums would go back to work part-time...the male psyche was to get into the profession, any profession, and you've got to provide for your family...and sometimes you've got that trap of, okay, now you've got that income coming in but you're burning out, because its, you know, 'God it's bloody Monday again tomorrow, I hate it'. But you're there because at the end of the month it's payday. *(Metropolitan Focus Group, New South Wales)

### The role of education and training

The relative status attached to the universities at which the different podiatry and physiotherapy courses were based tended to suggest a status differential; with some physiotherapy programmes taught at the more traditional 'sandstone' universities (Universities of Sydney, Melbourne and Queensland) as well as the newer universities (La Trobe University), whilst in the eastern states podiatry has been exclusively taught in the newer universities (the University of Western Sydney, La Trobe University and Queensland University of Technology). The status differential was felt to be particularly marked in New South Wales;

*University of Western Sydney is the lowest ranked university in New South Wales, and if nothing else that's going to affect our status as well. We would love to get podiatry to New South Wales or Sydney [universities] to raise the profile and get it out of Campbelltown, or to get it closer to Sydney. If I did podiatry now, I wouldn't travel to where it's being done to be a podiatrist. It's as simple as that, no matter how good it [the course] was...*(Telephone interview, New South Wales)

...*the status of the university [UWS] is way down on, say, Sydney University, and therefore the profession may be way down...It's a big thing. *(Telephone interview, New South Wales)

## Discussion

This paper has highlighted the complex nature of professional status as a subjective evaluation, and how it is intertwined with professional self-determination and autonomy.

The perceived key determinants of professional status broadly reflected those factors considered relevant within the wider sociological literature, notably educational credentials [1,34,48], client group status [4,40,49] the actual nature of the content and context of work [42,50], and the importance of a supportive 'ideological covering' in persuading different audiences to acknowledge a professional status [39,40]. In terms of the content of work, the development of 'virtuoso' roles was especially evident within 'specialised' public sector work, consisting of tasks requiring high levels of knowledge and technical skill.

Elevated status was felt to be associated with higher levels of specialisation at both an individual level and at the level of the profession collectively. Low status, conversely, was widely held to result from an association with the mundane tasks of corn and callus removal – identified by Farndon [18,19] in the UK as 'core podiatry' – and an essentially elderly client group. In order to change public perceptions, a perceived need to engage in marketing, both individually and collectively, was viewed as important. Interestingly, the marketing mechanism deployed to provide an image of specialisation seemed to be regarded as more important than the actual number of specialist interventions undertaken.

An expanded scope of practice was also felt to enhance status, where the tasks adopted were previously within the domain of medicine, whilst the loss of 'core' work to nurses and physiotherapists was viewed as a threat to professional identity, reflecting both the exclusionary and usurpationary basis of the stratagems of professionalism (the former refers to strategies operated by powerful groups, to ensure the exclusion of less powerful groups from key privileges; the latter constitutes the strategies adopted by the less powerful group in attempting to seize the privileges of a more powerful group). In this instance, the health policy demands for increasing workforce flexibility present both opportunities and challenges to the autonomy and status of the profession. Whilst enhanced roles in rural practice, due to the unavailability of medical care, led to the adoption of more medical tasks and a perceived increase in status, the loss of specialised and core activities to other, competing professional groups was felt to undermine it.

Importantly, the State, as a regulator, still plays a significant role in shaping the professional boundaries of podiatrists. This study identified two areas in which the scope of practice of podiatry was limited by state regulation (in some states); the practice of podiatric surgery, and access to prescription only medicines. Foot surgery is seen as one of the higher status aspects of podiatric practice, but remains limited to a small proportion of the profession. Barriers to prescribing [51] reinforce the dependence of podiatry on the medical profession for technologies which help in the treatment of common foot conditions (eg fungal infections of the skin).

Freidson's [48] assertion, echoing Weber's, that economic and cultural aspects of status are only 'modestly related' was reflected by the disparity between income and status evident in the public-private sector divide. Private sector podiatrists, although capable of securing greater income than their public sector colleagues, enjoyed less status within the profession. Yet the culture within the private sector was less professionalised, being more focused on the value of entrepreneurial skill and business acumen than higher professional status. Nevertheless, the status differential was acknowledged within both groups.

In terms of the perceived means by which effective change might be achieved, marketing and the active engagement of the professional body was viewed as central to the persuasion of the state, medicine and other powerful elites, whilst the individual practitioner was felt to be capable of advancing the status of the profession through the projection of an image which conveyed the complexity of podiatric tasks and responsibilities, by focusing on the more glamorous aspects of practice. Active capture of new, younger client groups, and the enhancement of scientifically credible knowledge and skills, manifest through academic qualifications, was also viewed as important. As Macdonald [36] noted, the 'respectability' of the institution within which the professions are educated provides a further mark of relative professional status. Training for podiatrists in Australia is typically provided at newer universities, with one notable exception (University of Western Australia), whilst physiotherapy training, for example, is provided by older, more established universities in Sydney, Brisbane and Melbourne, and these are generally seen as being more prestigious and are associated with medicine – a view which was expressed by several of the respondents.

Health care professions in many Anglophone countries are facing a period of enormous change, which has the potential to influence levels of professional autonomy and scope of practice. Indeed, many of the changes are contrary to the nature of the professional project itself and will potentially result in a reconfiguration of the health workforce which may structurally alter the professions.

Under the current model of professionalism, the success of the professions at achieving and sustaining a relative advantage and market share is likely to depend on their ability to maintain a monopoly over key aspects of their work; to persuade the public and other purchasers or funders that they are the best, or only, provider of particular aspects of health care; and to continue to attempt to diversify and potentially subsume aspects of work traditionally undertaken by other health care providers. At the same time, podiatry appears to be vulnerable to boundary encroachment by other professions (eg nurses and care assistants), and this requires the use of innovative measures to resist it. The adoption of internal closure strategies such as the deployment of subordinate grades in the form of 'foot care assistants', able to undertake the more mundane aspects of podiatric care, remains a contested issue in Australia, although an equivalent grade exists in the UK without affording clearly identifiable status benefits [16]. Australian podiatrists' views of their professional status does, then, shed light on the challenges that lie ahead for this allied health profession.

## Limitations of the study

This study is based on the perspectives of a sample of Australian podiatrists. Whilst we endeavoured to ensure appropriate diversity in the respondents, the largely self-selecting sample means that it is difficult to guarantee that the views of the participants of this study reflect the wider population of podiatrists. The convergence of views, and data saturation over several key points relating to status suggests that the views obtained by our study sample are an accurate representation. In addition, a copy of this manuscript was sent to a number of podiatrists across each participating state in Australia to determine whether it confirmed the perspectives of the interviewees, and to further verify the theoretical generalisability of our findings.

## Consent

All participants provided written consent for publication of the findings of this study research. A copy of the written consent is available for review by the Editor-in-Chief of this journal.

## Competing interests

AMB is Deputy Editor in Chief (UK) of the Journal of Foot and Ankle Research. It is journal policy that editors are removed from the peer review and editorial decision making processes for papers upon which they appear as co-authors.

## Authors' contributions

WV initially conceived the study and recruited the research team. Data was collected by AMB, SN, JW and WV. AB and SN analysed and interpreted the data. AMB and SN drafted the manuscript. All authors read and approved the final version of the manuscript.
